# Anacardic Acid (Exempted Substances*^1^) (Feeds, Fertilizers, etc.)

**DOI:** 10.14252/foodsafetyfscj.D-25-00019

**Published:** 2025-06-27

## Abstract

Food Safety Commission of Japan (FSCJ) conducted a risk assessment of anacardic acid (CAS No. 11034-77-8) in response to the request from the Ministry of Health, Labour and Welfare (MHLW) in line with the application for a new designation of cashew nutshell liquid (CNSL) as a feed additive. Anacardic acid, a major substance of CNSL, is an alkyl phenol suppressing methane production in the first rumen of cattle. Submitted documents for the feed additive designation was used for the current evaluation. The data used in the assessment include fate in animals (cattle and others), tissue residues (cattle), genotoxicity, acute toxicity (mice), and subacute toxicity (mice). FSCJ determined the non-observed-adverse-effect level (NOAEL) of 600 mg/kg bw per day for the females and 1,000 mg/kg bw per day (the maximum dose) for the males. Although chronic toxicity and carcinogenicity studies for anacardic acid have not conducted, possible concerns of chronic effects would be anticipated from the results of the subacute toxicity study, and also considering the low residue of anacardic acid and the long-term food experience of cashew nuts containing the anacardic acid. In Japan, CNSL containing anacardic acid has been applied as a feed ingredient to livestock since 2012. No safety issue has been reported on livestock or their products due to this feed ingredient. FSCJ concluded that anacardic acid would not harm human health through the residues in food as long as used ordinally as a feed additive.

## Conclusion in Brief

Food Safety Commission of Japan (FSCJ) conducted a risk assessment of anacardic acid (CAS No. 11034-77-8) in response to the request from the Ministry of Health, Labour and Welfare (MHLW) in line with the application for a new designation of cashew nutshell liquid (CNSL) as a feed additive. Anacardic acid, a major substance of CNSL, is an alkyl phenol suppressing methane production in the first rumen of cattle. Submitted documents for the feed additive designation was used for the current evaluation.

The data used in the assessment include fate in animals (cattle and others), tissue residues (cattle), genotoxicity, acute toxicity (mice), and subacute toxicity (mice).

The substantial non-absorption of anacardic acid from alimentary tract and fecal excretion without degradation were indicated from the data of cattle fed diet containing CNSL. Furthermore, alkyl phenols including anacardic acid were not detected in either tissues or milk, FSCJ recognized that anacardic acid administered would not be remained in the cattle.

FSCJ judged anacardic acid as non-genotoxic, considering the validity of negative results of *in vivo* genotoxicity study on anacardic acid and both *in vitro* and *in vivo* genotoxicity studies on CNSL and also cashew nutshell extract (CNSE).

Toxicity related to blood and kidneys was not observed in a subacute toxicity study up to the administration of 600 mg/kg bw per day. Female mice only experienced the toxicity after being administered 1,000 mg/kg bw per day, while the males were unaffected. FSCJ thus determined the non-observed-adverse-effect level (NOAEL) of 600 mg/kg bw per day for the females and 1,000 mg/kg bw per day (the maximum dose) for the males.

Although chronic toxicity and carcinogenicity studies for anacardic acid have not conducted, possible concerns of chronic effects would be anticipated from the results of the subacute toxicity study, and also considering the low residue of anacardic acid and the long-term food experience of cashew nuts containing the anacardic acid.

Reproductive and developmental toxicity studies for anacardic acid have not been conducted. No toxic effects on reproductive function or on the next generation have been reported in the long-term food consumption of cashew nuts containing anacardic acid. The residue level of this substance was expected to be negligible in the body as described above.

In Japan, CNSL containing anacardic acid has been applied as a feed ingredient to livestock since 2012. No safety issue has been reported on livestock or their products due to this feed ingredient. Alkyl phenol substances, such as anacardic acid, were not detected from the tissues or milk after the administration of this mixed feed additive to the cattle. FSCJ thus recognized that humans would unlikely take excessive amounts of anacardic acid derived from feed additives through food.

Given the above, FSCJ concluded that anacardic acid would not harm human health through the residues in food as long as used ordinally as a feed additive.
